# Performance Evaluation and Interference Characterization of Wireless Sensor Networks for Complex High-Node Density Scenarios

**DOI:** 10.3390/s19163516

**Published:** 2019-08-11

**Authors:** Mikel Celaya-Echarri, Leyre Azpilicueta, Peio López-Iturri, Erik Aguirre, Francisco Falcone

**Affiliations:** 1 School of Engineering and Sciences, Tecnologico de Monterrey, Monterrey, NL 64849, Mexico; 2 Electric, Electronic and Communication Engineering Department, Public University of Navarre, Pamplona, 31006 Navarra, Spain; 3 Institute of Smart Cities, Public University of Navarre, Pamplona, 31006 Navarra, Spain

**Keywords:** wireless sensor networks, interference characterization, performance evaluation, 3D ray launching, high-node density, smart cities

## Abstract

The uncontainable future development of smart regions, as a set of smart cities’ networks assembled, is directly associated with a growing demand of full interactive and connected ubiquitous smart environments. To achieve this global connection goal, large numbers of transceivers and multiple wireless systems will be involved to provide user services and applications anytime and anyplace, regardless the devices, networks, or systems they use. Adequate, efficient and effective radio wave propagation tools, methodologies, and analyses in complex indoor and outdoor environments are crucially required to prevent communication limitations such as coverage, capacity, speed, or channel interferences due to high-node density or channel restrictions. In this work, radio wave propagation characterization in an urban indoor and outdoor wireless sensor network environment has been assessed, at ISM 2.4 GHz and 5 GHz frequency bands. The selected scenario is an auditorium placed in an open free city area surrounded by inhomogeneous vegetation. User density within the scenario, in terms of inherent transceivers density, poses challenges in overall system operation, given by multiple node operation which increases overall interference levels. By means of an in-house developed 3D ray launching (3D-RL) algorithm with hybrid code operation, the impact of variable density wireless sensor network operation is presented, providing coverage/capacity estimations, interference estimation, device level performance and precise characterization of multipath propagation components in terms of received power levels and time domain characteristics. This analysis and the proposed simulation methodology, can lead in an adequate interference characterization extensible to a wide range of scenarios, considering conventional transceivers as well as wearables, which provide suitable information for the overall network performance in crowded indoor and outdoor complex heterogeneous environments.

## 1. Introduction

The notion of a smart world, with the aid of smart devices, smartphones, smart cars, smart homes, and smart cities, the paradigm of smart everything, has been a vigorously researched topic for many years. This concept holds the view that people and the world itself will be overlaid with sensing and actuation, with the aid of the internet of things (IoT). Nowadays, IoT has been used in a large number of areas, such as government, industry, and academia [1], for different applications. For example, sensors are placed in buildings for attempting to save energy [2,3]; wireless sensor networks (WSNs) in vehicular communications trying to improve safety and transportation [4]; home automation [5]; industry [6]; or e-Health services which are relying on increased home sensing to support remote medicine and wellness [7]. 

Nowadays, more than half of the world’s population lives in cities [8] with more than six devices per person connected to the Internet [9]. That means that billions of devices will be connected by 2020 to build the aforementioned smart city concept, which can range from end-user devices or wearables to vehicular communication systems, water and gas monitoring, smart lightning, structural monitoring, or smart healthcare systems, among others [10]. These solutions involve a high-density node environment, which in turn requires smaller outdoor and indoor cells leading to heterogeneous networks (HetNet). 

Moreover, the advent of next generation 5G communication systems implies the use of ultra-dense small cells in order to increase coverage/capacity requirements inherent to the wide array of services to be offered [11]. In this context, multiple wireless communication systems can be employed in order to diversify service provision, leading to issues such as unpredictable interference sources, or idle cell interference which can greatly impact quality of service [12,13]. Interference analysis has also been considered as a potentially beneficial evaluation element that can be employed in order to enable covert communications in IoT scenarios [14]. 

The complexity in interference analysis is given by multiple factors, such as requested service types, heterogeneous service requirements, wireless system coordination, or user location and density. In this sense, HetNet architectures implement superimposed cell structures in order to provide adequate capacity requirements as a function of the requested service type. However, this has a serious impact in overall interference levels, requiring the use of cell coordination, such as almost blank subframe technique in order to minimize transmission times [15], employing game theory approaches to provide first order approximation to consider overall interference levels or a similar approach in order to analyze multi-user-multiple-input-multiple-output (MU-MIMO) user assignment in 5G systems [16]. Not only do user distribution and service type have an impact on interference, but also hardware constraints, such as sampling rate mismatch between end user devices and access points/base stations, also affect in the case of massive IoT deployments [17]. In this same line, interference levels can degrade operation of massively deployed transceivers, such as LoRa, owing to loss of ideal orthogonality and leading to increased packet loss [18]. In the future, these constraints can further be aggravated by the use of novel schemes, such as radio frequency (RF) energy harvesting, which in the case of IoT deployments, must consider coverage/capacity relationships in order to comply both with minimum harvested energy thresholds as well as with uplink/downlink signal to interference ratios [19]. Hence, interference analysis and control are one of the fundamental aspects to consider in the scenarios with variable quality of service (QoS) requirements and user density and location, intrinsic to IoT applications [20]. Different solutions have been recently proposed in order to control overall interference levels, such as content-aware cognitive control [21], resource split full duplex mechanisms [22], or machine learning techniques to provide adaptive transmit power control in WSNs [23].

For the successful implementation of the aforementioned dense node deployments, reliable and accurate channel models are necessary, addressing the different topologies and radio links to get reliable service, coverage, and capacity, as well as interference management. Furthermore, hot spots have a non-uniform traffic demand, so it is necessary to have three-dimensional (3D) realistic environments to achieve accurate models, which can lead to network performance improvement. The approaches followed in order to analyze interference in large areas with node density are usually based on statistical channel modelling and under certain model assumptions [13,24], providing certain consideration in relation with scenario characteristics, which can be eventually combined with measurement updates. Spatio-temporal techniques have also been proposed in order to analyze connection establishment phases in massive IoT deployment, based stochastic geometric models [25]. Measurement based interference characterization in IoT scenarios has also been proposed, assisted by supervised learning [26]. However, none of these approaches perform a complete analysis and system performance evaluation considering the whole morphology and topology of the considered scenario. Moreover, realistic wireless system operation exhibits a complex behavior, depending on conditions such as household/office environment, wireless systems under operation within the scenario under analysis or the density of transceivers considered [27]. 

The analysis on wireless node density and variations within wireless channel characteristics is relevant in functionalities related with applications such as wireless cooperative location systems [28] or in passive location systems [29], in which wireless channel characteristics (line of sight-non-line of sight channel conditions as well as multipath propagation) influence ranging estimations and hence, location performance. Network synchronization is another application in which node density impacts system operation of cooperative systems, influencing the value of the cooperative dilution intensity [30], which is influenced by wireless channel conditions. 

Energy analysis is a relevant aspect in the operation of wireless communication systems and particularly in wireless sensor networks, with the existence of inherent limitations given by restrictive energy sources, processing capabilities, and compact form factor requirements. In this sense, wireless sensor network energy balance analysis is compulsory in order to implement efficient system level solutions, such as scheduling algorithms for sleep/active states in order to implement wireless sensor networks operating under partial coverage conditions [31]. By studying required coverage levels (given by receiver sensitivity thresholds, determined by transmission bit rates, adaptive modulation and coding schemes, and electronic device characteristics), transceivers can be dynamically set in sleep modes, resulting in effective energy reduction and hence, enhanced operation lifetime. In this context, estimation of wireless channel behavior, in terms of coverage estimation as well as in distribution of interference sources is relevant in order to analyze overall energy consumption impact, from physical layer as well as in access control and network layer.

In this work, we present a deterministic technique to model electromagnetic propagation in high node density scenarios, specifically an in-house 3D ray-launching (3D-RL) algorithm, based on geometrical optics (GO), geometrical theory of diffraction (GTD), and its extension the uniform theory of diffraction (UTD). With the aid of the 3D-RL simulation tool, the performance evaluation and interference characterization of a dense node density scenario has been performed in order to assess the key performance indicators of the network. The contributions of this work are aimed in providing a precise tool for coverage/capacity estimation, considering relevant multipath propagation phenomena in large complex scenarios. The proposed simulation methodology employs an optimized 3D-RL code with hybrid simulation (combining 3D-RL with neural network interpolators, the electromagnetic diffusion equation for diffraction estimation, and collaborative filtering of deep learning data base algorithms), enabling the possibility to simulate large, complex scenarios. A new simulation module has been implemented in order to perform estimation of error vector magnitude (EVM) for the complete simulation volume, hence enabling further quality of service analysis as a function of the employed modulation scheme. 

The remaining parts of the paper are outlined as follow: The proposed simulation technique and the scenario description are explained in Section 2. Section 3 presents the simulation results in the high node density considered scenario, at ISM 2.4 GHz and 5.8 GHz frequency bands, with the received signal strength (RSS), signal to interference noise ratio (SINR), and performance analysis in terms of constellations plots and EVM characterization considering a ZigBee system (infrastructure nodes) as well as Bluetooth transceivers (high mobility devices/users), and coverage/capacity estimations examples. In Section 4, a campaign of measurements has been presented for the same considered scenario, achieving a good match between simulation and measurement results. In addition, the comparison between the scenario full of people and without people is presented in this section. Finally, conclusions and future work are summarized in Section 5. 

## 2. Proposed Simulation Technique

### 2.1. The RL Technique

The in-house developed 3D-RL simulation tool has been developed in Matlab programming environment. The detailed operating mode of the algorithm has been previously published [32] and validated in complex urban environments [33]. The principle of the RL approach is to approximate the full wave methods based on Maxwell’s equations into a set of equations based on GO and UTD. The in-house implemented 3D-RL code has been optimized in order to decrease computational cost when consider large, complex scenarios, such as indoor locations, by means of hybrid simulation approach. In this sense, neural network interpolators, electromagnetic diffusion equation for diffraction estimation, and deep learning database assistance by means of collaborative filtering have been implemented within the code. The algorithm basis has three steps:

- Creation of the 3D environment.
- Simulation procedure.
- Results analysis.

The first step consists in the creation of the 3D environment under evaluation. For that purpose, all the details of the environment are considered, taking into account its real dimensions, morphology, topology, and material properties (by means of the conductivity and dielectric permittivity) for all the obstacles within the scenario at the frequency band under analysis. In the simulation procedure, a set of rays are launched from the transmitter and electromagnetic propagation phenomena such as reflection, refraction, and diffraction are considering along all the path rays. Parameters such as transmitters location, angular resolution of rays, cuboids size of the scenario, frequency of operation, and number of reflections are considered as input parameters in the algorithm. A trade-off between angular resolution of launching rays, cuboids size of the scenario, required computational time, and results accuracy must be achieved during the simulations. A convergence analysis in terms of number of reflections and launching rays of the algorithm has been performed and it is presented in [32], as well as the optimal spatial resolution for large scenarios, which is presented in [34]. These parameters are used in the simulations and are presented in Section 2.2. 

Finally, the third step consists in the results analysis, where different outcomes can be obtained. The 3D-RL tool is based on a modular structure, where the user can select the results of interest. The different results that can be selected are large-scale propagation parameters such as received power or path loss analysis, or small-scale parameters such as power delay profile (PDP), delay spread, coherence bandwidth, or doppler spread, among others. In this work, interference analysis has been implemented as a new module for the network performance assessment. In this library, once the power level and signal to interference noise ratio (SINR) results have been obtained for all the spatial points of the scenario, different modulations can be assessed for different communication links, presenting evaluation of EVM within the complete volume of the scenario under analysis. 

### 2.2. Scenario Description

**Figure 1:**
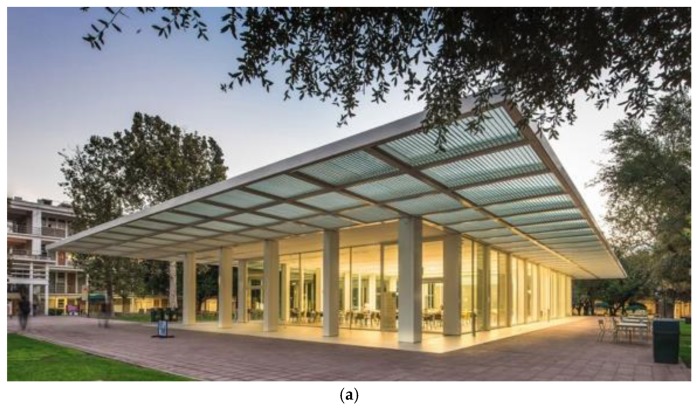

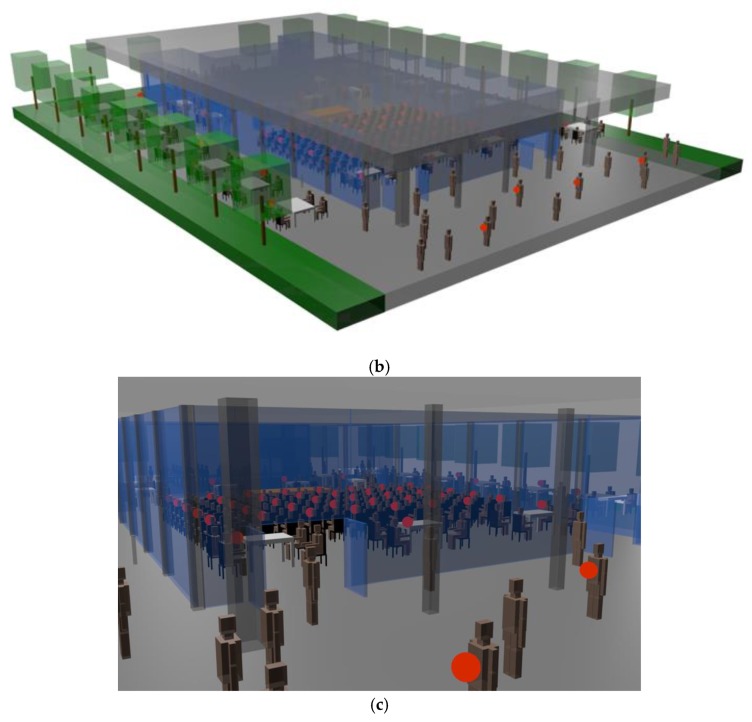
Urban considered scenario where, (**a**) is the real view of the scenario (retrieved from: http://www.rdlparquitectos.com/es/proyectos/pabellon-la-carreta/), (**b**) is a 3D aerial rendered view, and (**c**) detailed view of the indoor part of the scenario.

The selected scenario is an auditorium placed in an open free city area surrounded by inhomogeneous vegetation. Figure 1 presents the real and schematic view of the considered scenario, which is part of the Campus of Tecnologico de Monterrey, Monterrey, Mexico. The considered scenario is a complex scenario in terms of radio wave propagation characterization as it is a combination of an outdoor and indoor environment rich in multipath trajectories due to the large quantity of obstacles and people within it. The different workspaces of the auditorium environment have been recreated in the simulation algorithm, taking into account the inhomogeneous vegetation, trees, tables, chairs, the auditorium area, the cafeteria area, corridors, and a random distribution of people, both in the outdoor and indoor areas of the auditorium. A generic human body model design created specifically to be embedded in the 3D-RL algorithm has been used to enhance a more realistic scenario, as users have a significant influence in radio wave propagation in this type of complex environments [35]. The specific details of the developed human body model and its integration with the 3D-RL tool can be found in [36]. 

**Figure 2:**
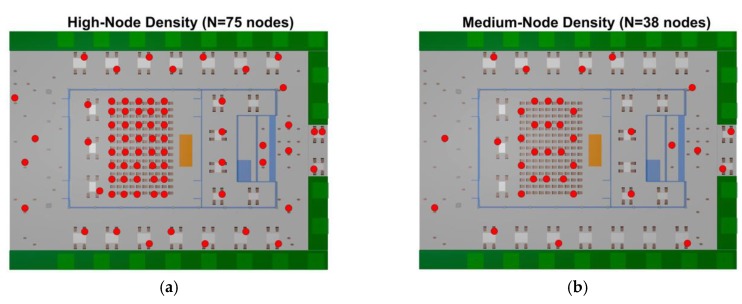

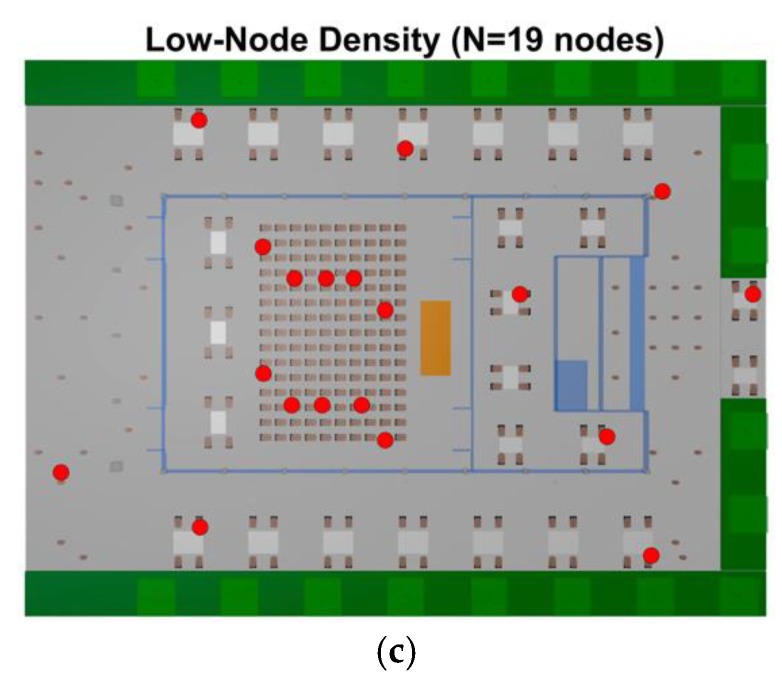
Aerial view of the scenario with the wearable’s location for the three different node density cases. Auditorium ceiling has been removed for illustration purposes, (**a**) high-node density, (**b**) medium-node density, and (**c**) low-node density.

User density within the scenario, in terms of inherent transceivers density, has been performed by simulation. The considered scenario has a user capacity of 150 persons within the auditorium, and 40 more approximately, when all the tables around the auditorium are occupied. Thus, for a high-node density within the complete scenario, it has been considered that one per four persons has a wearable that can increase overall interference levels, which lead to a sensor network of 75 wearables. A medium node density has been considered with 38 wearables (one device per eight persons), and a low node density of 19 wearables (one wearable over 16 persons). All the wearables have been considered at 1.2 and 0.8 m height, emulating smart glasses or wrist-worn devices in the case of seated persons. Figure 2 represents an aerial view of the scenario (ceiling has been removed for illustration purposes) with the wearable’s location for the three different nodes density cases. For the simulations, two different frequencies have been considered, 2.4 GHz and 5.8 GHz, considering the later for infrastructure operation (i.e., not as wearable devices). For the network performance analysis, a ZigBee system has been considered at 2.4 GHz frequency band, as well as Bluetooth V4.0 transceivers within the scenario. This election is based on two modes of operation: Data gathering (from WSNs or users) and wireless transport networks (given by 5.8 GHz WLAN devices). ZigBee offset-quadrature-phase-shift-keying (O-QPSK) modulation, with a bandwidth of 3 MHz and a bit rate of 250 kbps, whilst Bluetooth V4.0 employs at higher rates, differential 8-level phase-shift keying (8-DPSK) modulation at a bit rate of 3 Mbps. Simulation parameters are summarized in Table 1. 

## 3. Simulation Results

### 3.1. Received Signal Strength

**Figure 3:**
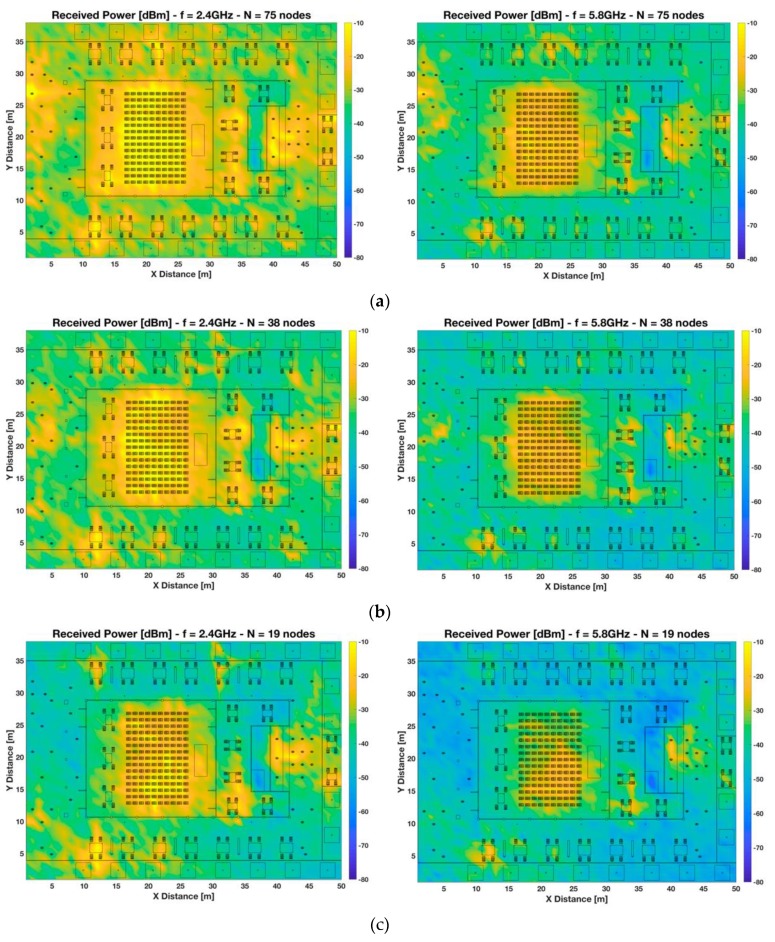
Bi-dimensional planes of received power [dBm] at 1.2 m height at 2.4 GHz and 5.8 GHz frequency bands. Different scenario setups have been considered: (**a**) high-node density scenario, (**b**) medium-node density scenario, and (**c**) low-node density scenario.

**Figure 4:**
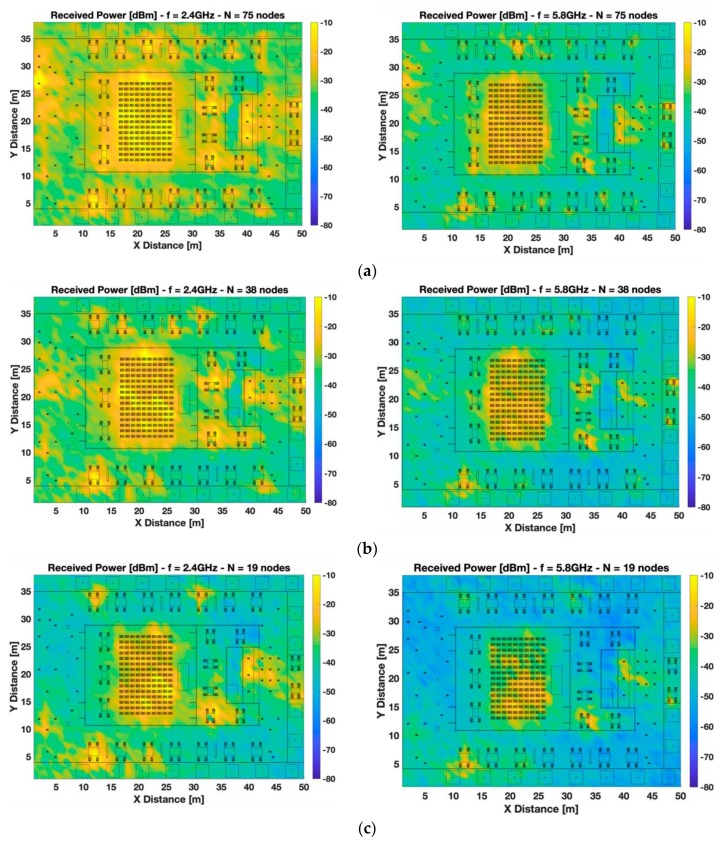
Bi-dimensional planes of received power [dBm] at 0.8 m height at 2.4 GHz and 5.8 GHz frequency bands. Different scenario setups have been considered: (**a**) high-node density scenario, (**b**) medium-node density scenario, and (**c**) low-node density scenario.

Firstly, the Received Signal Strength for the full volume of the scenario has been obtained by means of the 3D-RL simulation tool. Although the whole volume of the scenario has been analyzed in simulation, only the bi-dimensional planes of received power for a selection of relevant heights have been presented in Figure 3 and Figure 4. The considered heights have been 1.2 m, which is the same height as the transmitters, emulating smart glasses receivers for a seated person (Figure 3), and 0.8 m height, emulating wrist-worn receivers for a seated person (Figure 4). 

Figure 3 presents the RSS maps at 1.2 m height considering different node density setups for two different frequencies, 2.4 GHz and 5.8 GHz, which are the typical frequencies used in generic wearables, as well as in wireless sensor network infrastructure (i.e., 802.15.4 networks and 802.11 networks). It can be seen that the morphology and topology of the scenario, as well as node density distribution, have a great impact in radio wave propagation. It is observed a high RSS variability within the whole scenario due mainly to the presence of different scatterers such as people, furniture, walls, vegetation, and trees, among others, which cause a rich multipath propagation in the environment under analysis. It can also be remarked that RSS is around 20–30 dBs higher when high-node density is considered. Moreover, it is worth noting that there are localized regions with higher power levels, i.e., hot spots, within the scenario, caused by individual transceivers, that tends into power clusters when the number of nodes increases in a region. This is the case of the auditorium indoor area, where there is a higher concentration of wearables, and therefore it also has a higher concentration of received power (around 5–10 dB more), being more remarkable in the case of high-node density due to the higher number of wearables involved. The higher intensity in this area is also given by the body shielding effect, which enhances the signal to be concentrated in the auditorium indoor area, where more users are presented. In addition, different regions can be identified within the simulation scenario where power levels differ, such as the outdoor and indoor part of the scenario, or the cafeteria area in the right part of the scenario. These regions are delimited by different types of walls (glass in the auditorium and concrete/metal in the cafeteria), which gives rise to different associated propagation phenomena, such as the appearance of hot-spots in the aforementioned localized areas, regardless node density setups. Frequency also plays an important role showing that for all the analyzed cases, higher power levels are received at lower frequencies, as it was expected. The presence of hot-spots is also more remarkable at lower frequencies (notice that the power level scale has been maintained for both frequencies for better comparison). 

Figure 4 presents the same results as Figure 3, but in this case, for a 0.8 m height, emulating wearables such as smart watches operating in the wrist of a seated person. As it can be seen, a high variability of RSS is also observed in this case, showing slightly lower values of received power because the bi-dimensional plane representation is not at the same height of the transmitter’s wearables. In these cut-planes, the presence of hot-spots is still visible but with lower intensity for all the different node density setups. This specific behavior is caused due to bigger link distances with the transmitters comparing with the previous cases analyzed. Besides, there are relevant differences between the operating frequencies, with more losses at the higher frequency, as it was expected.

**Figure 5:**
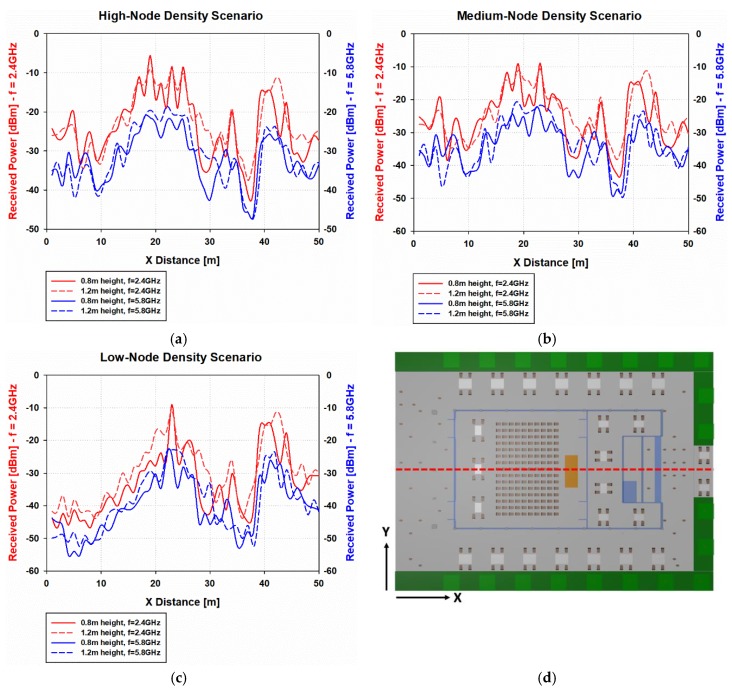
Linear distribution of received power [dBm] for different heights and frequency bands for Y = 20 m along the X-axis, (**a**) high-node density scenario, (**b**) medium-node density scenario, (**c**) low-node density scenario, and (**d**) radial line representation.

In order to gain a better insight into the differences of received power for different heights and frequencies in the three nodes density cases, the radial distribution of RSS in the considered scenario has been assessed and it is represented in Figure 5. The considered radial line is depicted in Figure 5d, a representation of the aerial view of the scenario (ceiling has been excluded for illustration purposes), which correspond to Y = 20 m along the X-axis. As it was expected, in the comparison for the low-node density case, the trend of the obtained received power values is lower than in the other cases. Hence, the distribution of received power levels depends strongly on the node’s distribution in the considered scenario. In addition, there is a slightly difference between RSS values at the different heights, showing higher values in general for the 1.2 m height (the same cut-plane as the transmitters). However, this trend is not homogeneous for all the spatial points, because as stated above, the morphology and topology of the scenario plays an important role in radio wave electromagnetic propagation for complex environments. Regarding the different frequencies, at the 5.8 GHz frequency band the losses are 5–10 dB approximately higher than the 2.4 GHz frequency band. 

### 3.2. Signal to Interference Noise Ratio

Effective signal and interference levels are determined by received power level distributions, which in turn are determined by the number of nodes as well as their topological distribution, in relation with the surrounding environment. In order to have clear insight into the interference levels in the proposed sensor network, SINR volumetric estimations have been obtained for the whole scenario. An acceptable communication link formation in terms of quality of service is given by the fulfillment of the following condition [37]:

(1) $$P_{RX}\left(\vec{d},RX_{hw}\right)\geq SENS_{RX}\left(SINR,\;R_{b},\;m_{c}\right)$$

where $P_{RX}\;$is the received power for each transceiver, as a function of spatial location $\vec{d}$ and receiver hardware parameters $RX_{hw}$ (e.g., antenna gain, noise factor) and $SENS_{RX\;}$is the receiver sensitivity, determined by the required SINR threshold (or $E_{b}/N_{0}\;$in the case of digital systems), transmission bit rate $R_{b}\;$and the modulation and coding scheme $m_{c}$. In this context, the determination of useful received power and detected interference levels provides coverage/capacity relations as a function of service requirements and density of nodes within the scenario. The scenario location of all transmitting, as well as receiving elements, is a fundamental parameter, due to the large variability in power distribution in the complex environment under consideration.

Once the wireless channel characterization has been performed for different transceiver densities, as described in previous section, interference analysis in terms of SINR can be obtained, leading to system coverage/capacity estimations. For that purpose, the worst-case conditions have been considered, in terms that SINR analysis are provided when the interconnecting device operates with in-band inter-system interference. For the different node density cases, one interconnecting device has been considered as the transmitter and the rest as in-band inter-system interference. 

**Figure 6:**
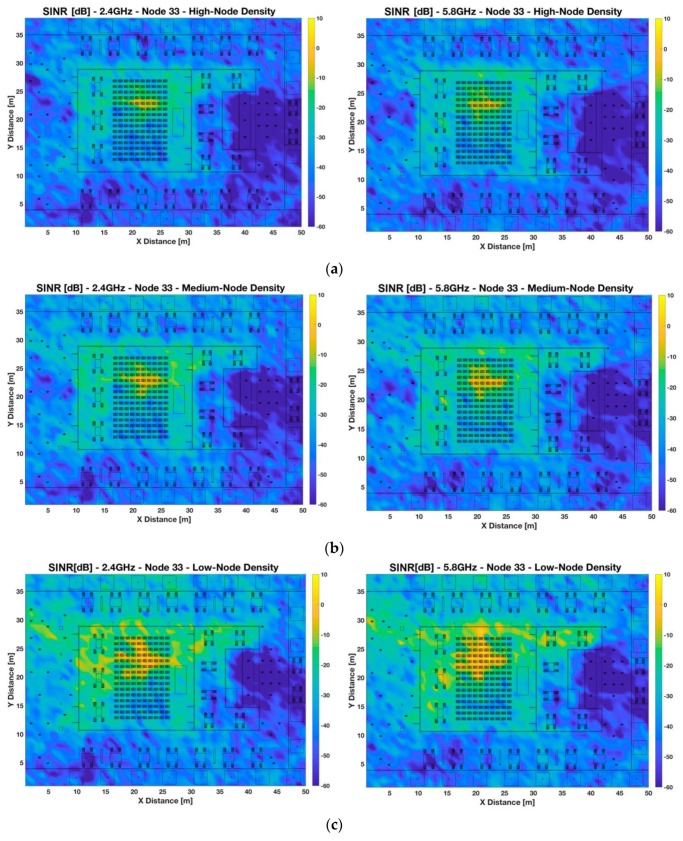
Bi-dimensional planes of signal to interference noise ratio (SINR) [dB] for Node 33 (wearable placed at 1.2 m height, same as a seated person in the indoor area of the auditorium, X = 20.35 m, Y = 22 m) at 2.4 GHz and 5.8 GHz frequency bands. Different scenario setups have been considered: (**a**) high-node density scenario, (**b**) medium-node density scenario, and (**c**) low-node density scenario.

**Figure 7:**
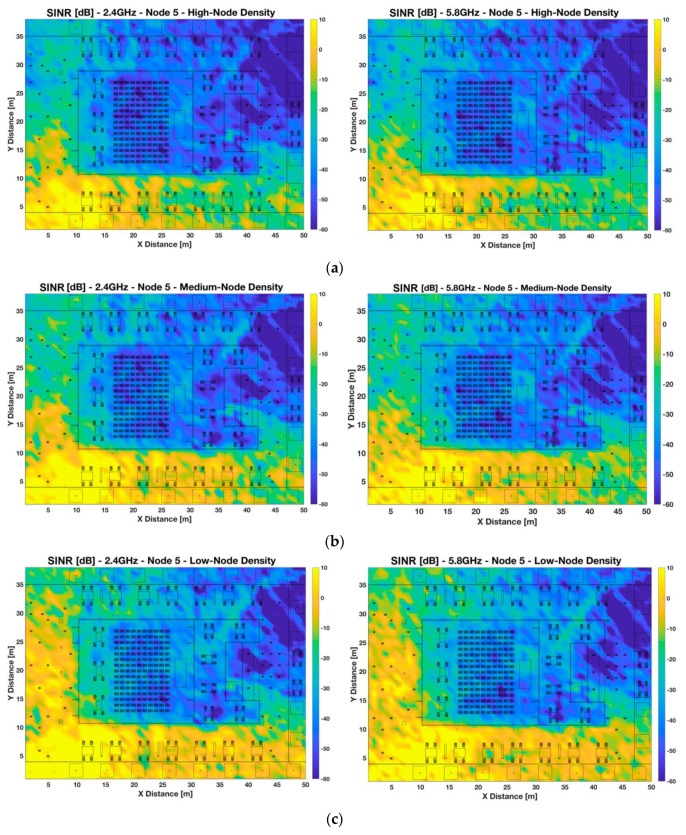
Bi-dimensional planes of SINR [dB] for Node 5 (wearable placed at 1.2 m height, same as a seated person in the outdoor area of the auditorium, X = 11.7 m, Y = 5.8 m) at 2.4 GHz and 5.8 GHz frequency bands. Different scenario setups have been considered: (**a**) high-node density scenario, (**b**) medium-node density scenario, and (**c**) low-node density scenario.

Figure 6 and Figure 7 show the bi-dimensional plots at 1.2 m height of the SINR distribution for two different locations of the transmitter, indoor and outdoor, considering different sensors densities distributed non-uniformly in the scenario, as interferers (see Figure 2 to have insight into the different node distribution densities). These plots represent an upper bound in relation with quality degradation in terms of simultaneous operation of the transceivers. The provided SINR values can be mapped afterwards to $E_{b}/N_{0}\;$ ratios, where modulation scheme as well as transmission bit rate can be explicitly considered.

Figure 6 presents the SINR values considering the transmitter a wearable (i.e., smart glasses) of a seated person inside the auditorium (X = 20.35 m, Y = 22 m) (i.e., Tx 33) and the simultaneous operation of the rest of transceivers in different node densities setups. It can be observed that the highest SINR values appear in localized areas nearby the transmitter in the indoor part of the auditorium. The differences between nodes densities are pronounced, around 10–15 dB approximately between the low- and high-node density cases for the indoor area of the scenario. Besides, for all density cases, the SINR values for the outdoor region of the scenario are very low. These results show the high dependence of node density and represent an upper bound in terms of quality degradation when other transceivers are operating simultaneously. Regarding the differences between different frequency bands, it can be seen that there is not a lot of variability in the SINR values between different frequencies, but for the 5.8 GHz frequency band, SINR values are slightly lower, depending of the spatial considered point due to the morphology of the scenario. 

To gain insight into the significance of considering the whole three-dimensional scenario, the same SINR analysis has been obtained for an outdoor located transmitter node, a wearable of a seated person placed in the outside tables of the auditorium, in the left down corner of the scenario (X = 11.7 m, Y = 5.8 m) (i.e., Tx 5). These results are presented in Figure 7, where a bigger area than the previous case of high values of SINR around the selected transmitter can be observed. This is caused because of the smaller number of scatterers presented in the outdoor part, which allows a higher area with high SINR values, for all different node density cases. Besides, the outdoor-indoor communication is deeply affected by the scenario boundaries, such as the auditorium walls, being the worst case for the high node density case. For all node density cases, it can be observed that the SINR values in the indoor area of the auditorium are very low, which means that the communication link will not be feasible with the indoor region of the auditorium. As in the previous case, there are not many differences in SINR values between frequencies, with slightly lower SINR values for the higher frequency band (5.8 GHz). 

### 3.3. Performance Analysis

Coverage analysis can aid in an adequate estimation of indoor and outdoor coverage in the simulated scenario. However, since coverage and capacity are linked, it is mandatory to analyze the influence of modulation in order to determine system performance. For that purpose, a ZigBee sensor network has been considered, which uses O-QPSK modulation, with a bandwidth of 3 MHz and a bit rate of 250 kbps, as well as a Bluetooth network considering V4.0 transceivers at a bit rate of 3 Mbps. As stated before, the worst-case conditions have been considered, in terms that for the different nodes density cases, one interconnecting device has been considered as the transmitter and the rest as in-band inter-system interference. 

**Figure 8:**
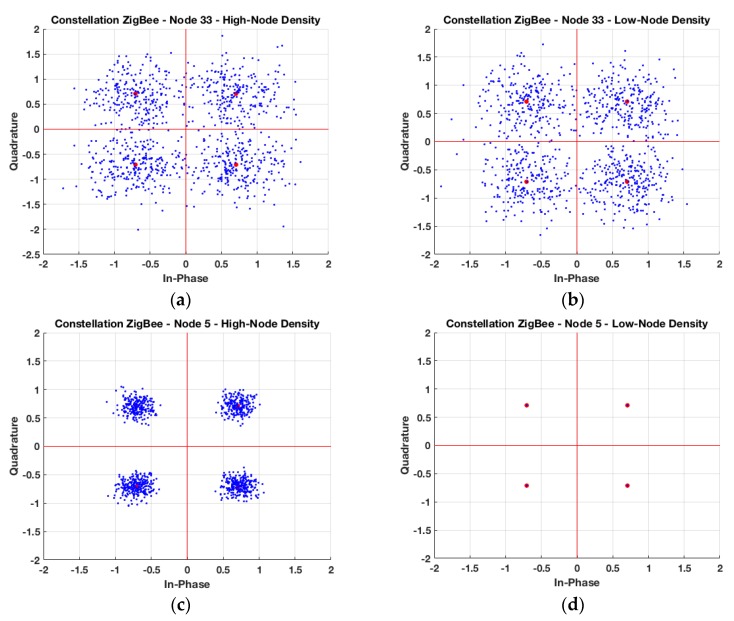
In Phase and Quadrature representation for two different nodes within the auditorium for offset-quadrature-phase-shift-keying (O-QPSK) modulation, (**a**) indoor node with high-node density scenario, (**b**) indoor node with low-node density scenario, (**c**) outdoor node with high-node density scenario, and (**d**) outdoor node with low-node density scenario.

Figure 8 presents the constellation plots at two different receiver location points placed in the indoor and outdoor area of the scenario for the ZigBee system. Two different transmitter positions have been considered, which are the same ones as the previously presented results: The first one in the indoor area of the scenario (Tx 33) and the second one in the outdoor area of the scenario (Tx 5). The considered receiver spots are placed at one-meter distance of each transmitter, in the indoor and outdoor area, respectively, considering high and low-node density. It can be seen that the symbols are more disperse in the case of the transceivers placed in the indoor part of the auditorium, for both density nodes. This is explained because simulations have been made with the auditorium full of people, which causes a high number of scatterers in the area. Thus, this coupled with the assumption of the worst-case condition, where all the nodes except the transmitter are in-band inter-system interference, causes a large dispersion in the received symbols in the indoor area of the scenario. Constellations plots for the receiver location point in the outdoor area of the scenario have less symbol dispersion, achieving the ideal constellation for the low-node density case, as the interferers in this case are not placed nearby the receiver. 

To have insight into the total interference, the error vector magnitude (EVM) has been calculated for the different node-density cases. The EVM is an indicator of the modulation accuracy. To quantify the modulation error, the amplitude of the error can be calculated as:

(2) $$Error\;Amplitude=\;\sqrt{{\left(I_{i}-I_{A}\right)}^{2}+{\left(Q_{i}-Q_{A}\right)}^{2}}=\sqrt{\Delta I^{2}+\Delta Q^{2}}$$

where $I_{i}$ and $Q_{i}$ are the In-phase and Quadrature values of an ideal signal, while the actual location of the signal is $I_{A}$ and $Q_{A}$. The root mean square (RMS) value of the error amplitude for *N* symbols is:

(3) $$RMS\;Error\;amplitude=\;\sqrt{\frac{1}{N}\left(\overset{N}{\underset{k=1}{\sum }}\Delta I_{k}^{2}+\Delta Q_{k}^{2}\right)}$$

If the ideal signal amplitude is *S*, the EVM is defined as:

(4) $$EVM\left(\%\right)=\frac{RMS\;Error\;amplitude}{Ideal\;Signal\;Amplitude}=\frac{\sqrt{\frac{1}{N}\left(\sum_{k=1}^{N}\Delta I_{k}^{2}+\Delta Q_{k}^{2}\right)}}{S}\times 100$$

Table 2 presents the EVM (%) for the constellations’ plots presented in Figure 8, including also the medium-node density case. The EVM requirement of the IEEE 802.15.4 standard must be less than 35% [38]. According to these results, the high-node density case for the indoor link is really close to the limit, so the received signal is going to be interference limited. 

**Figure 9:**
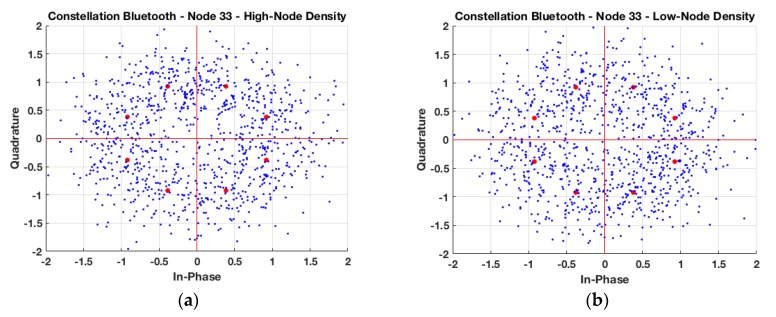

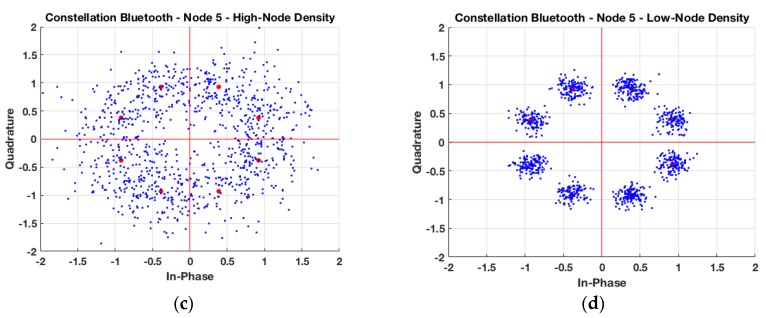
In Phase and Quadrature representation for two different nodes within the auditorium for 8-DPSK modulation, (**a**) indoor node with high-node density scenario, (**b**) indoor node with low-node density scenario, (**c**) outdoor node with high-node density scenario, and (**d**) outdoor node with low-node density scenario.

EVM analysis has also been performed in the case of considering operation of Bluetooth V4.0 transceivers within the scenario, a typical case for users or devices with high mobility. As in the previous case, variations in node density have a strong impact in EVM response, given by higher interference levels. These results are depicted in Figure 9, for the cases of node #33 and node #5, for different node densities. Table 3 presents the EVM (%) for the constellations’ plots presented in Figure 9, including also the medium-node density case.

**Figure 10:**
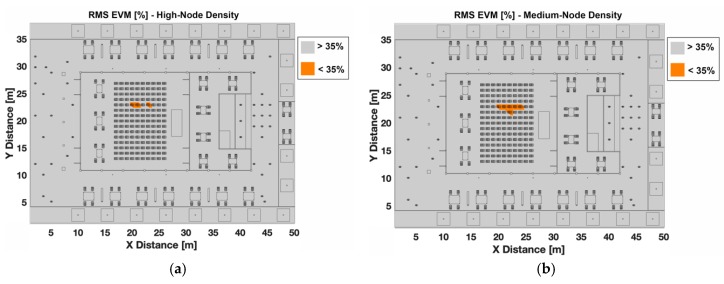

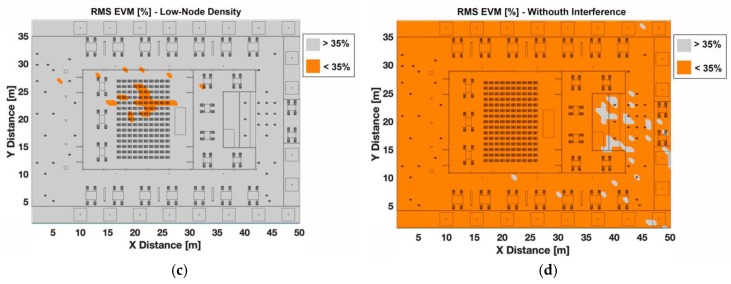
EVM (%) for the considered scenario for O-QPSK modulation when indoor node is transmitting, (**a**) high-node density scenario, (**b**) medium-node density scenario, (**c**) low-node density scenario, and (**d**) without interference.

To gain insight into the modulation error scenario characterization, the specific regions of correct operation regarding EVM for the ZigBee system have been mapped along the bi-dimensional cut planes. Although results have been obtained for the complete volume of the scenario, for the sake of clarity only the 1.2 m height has been depicted. Figure 10 presents the EVM for the different nodes-density cases and the case without interference, for a transmitter placed in the indoor area of the scenario (Tx 33) with again the worst-case conditions in terms of in-band inter-system interference. These operating regions are delimited by the configuration of the interfering network, as well as by the characteristics of the environment. It can be seen that interference levels can lead to have no service in different areas of the considered scenario, being these no-service areas bigger when high-node density is considered. It is worth noting that the low-density case considered is 19 nodes as it is presented in Figure 2c, which also implies a high interference level, as it can be seen in Figure 10c when compared with the case without interference (Figure 10d). 

**Figure 11:**
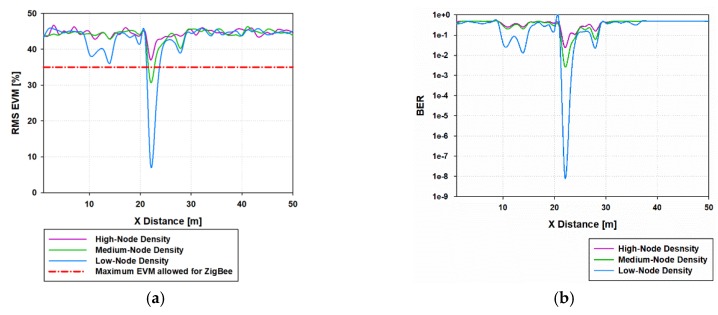
(**a**) Linear distribution line of EVM (%) for Y=20 m, X-axis in the considered scenario when indoor node is transmitting, (**b**) Linear distribution line of bit error rate for Y = 20 m, X-axis in the considered scenario when indoor node is transmitting.

Figure 11a presents the linear distribution line of EVM (%) for Y = 20 m along the X-axis in the considered scenario, for Tx 33 (indoor node X = 20.35 m, Y = 22 m), considering the three different node-density cases. There is only correct service in a small area around the transmitter because of the high interference levels which have been considered. Figure 11b presents the bit error rate (BER) for the same radial line for the three different node-density cases, showing that the BER is quite high in remote areas from the transmitter. 

**Figure 12:**
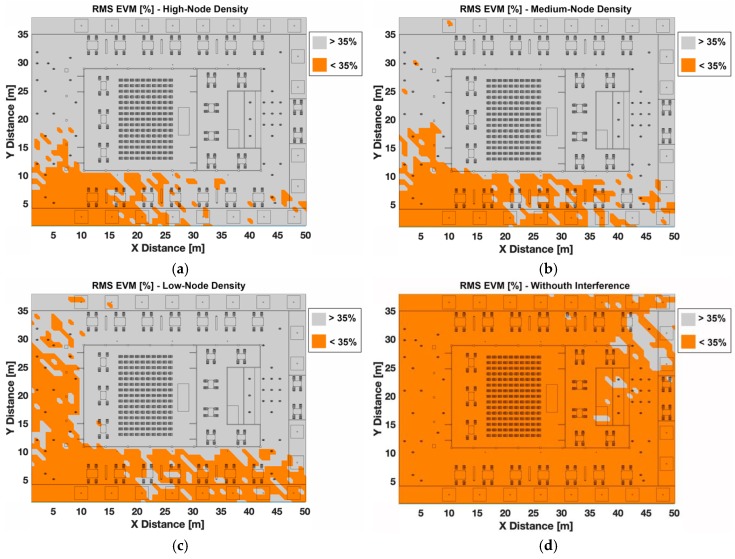
EVM (%) for the considered scenario for O-QPSK modulation when outdoor node is transmitting, (**a**) high-node density scenario, (**b**) medium-node density scenario, (**c**) low-node density scenario, and (**d**) without interference.

A second transmitter position has been considered to perform the EVM analysis in terms of O-QPSK modulation. In this case, the transmitter is placed in an outdoor location (Tx 5) at 1.2 m height. Regions of correct operation can be seen in Figure 12 for the different node-density cases and the case without interference. 

In comparison with the previous indoor transmitter case, the correct operation areas obtained are bigger in this case for all density cases, concentrating the valid area mostly in the outdoor part of the scenario. These results can be explained due to, as stated previously, operating regions are delimited by the boundaries of the scenario (i.e., walls) and the interfering network configuration. Besides, in the outdoor area of the scenario, there is less people, so the concentration of nodes is lower (see Figure 2 for reference), which increases correct operation area compared to the previous case. 

**Figure 13:**
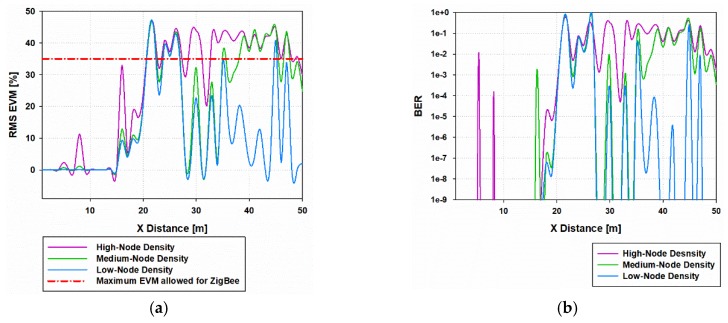
(**a**) Linear distribution line of EVM (%) for Y=5 m, X-axis in the considered scenario when outdoor node is transmitting, (**b**) Linear distribution line of bit error rate for Y = 5 m, X-axis in the considered scenario when outdoor node is transmitting.

Figure 13a presents the linear distribution line of EVM (%) for Y = 5 m along the X-axis in the considered scenario for Tx 5 (outdoor node X = 11.7 m, Y = 5.8 m) considering the three different node-density cases. It can be seen that in the vicinity area of the transmitter, correct operation for the three density cases is obtained, but as we move away from the transmitter, in the medium and high-node density cases, there is no system service due to higher interference levels. Figure 13b presents the BER of the same radial line for the three different node-density cases. As it was expected, BER is quite high in remote areas from the transmitter, and lower BER is encountered close to the transmitter. 

These results can aid in a better knowledge of the network performance and are relevant in terms of interference analysis as well as on the operation of mitigation schemes, when high-node density setups are presented. 

**Figure 14:**
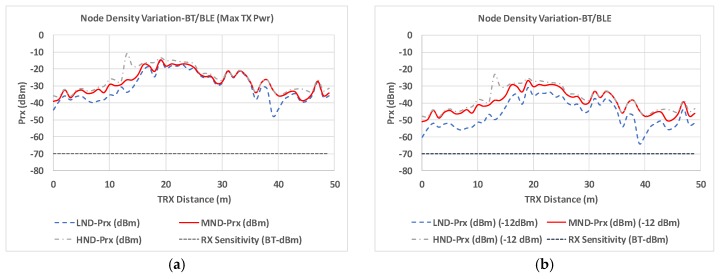
Coverage/capacity estimation for BT/BLE transceiver operation (**a**) max transmission power (4 dBm) (**b**) conventional transmission power (−12dBm).

Node density distributions have a direct impact in overall system performance. This can be observed in terms of coverage/capacity estimations, which depend on wireless system operating parameters (e.g., receiver sensitivity as a function of bit rate, employed adaptive modulation, and coding scheme). Coverage/capacity values can be precisely obtained by the use of 3D-RL simulation techniques, as they provide volumetric estimations of received power levels. As an example, estimations of coverage for receiver sensitivity in Bluetooth/Bluetooth Low Energy (BT/BLE) device distributions are depicted in Figure 14, as a function of transmission power levels. Two different situations are depicted: For maximum transmit power (4 dBm) and for conventional transmission power (usually set at −12 dBm). As a function of node density, variations in received power levels exhibit average received power level variations in the order of 4.8 dB, which can lead to coverage limit conditions (e.g., location at 38 m in the linear TRX radial for the conventional −12 dBm power consideration). 

From the previous results, performance is general degraded as node density increases. This is given by the fact that radio resource functionalities have not been considered, such as multiplexing strategies or dynamic frequency allocation. In this way, worst case operation conditions have been considered, as an initial bound in coverage/capacity analysis. Future work can be foreseen in the analysis of coverage/capacity relations as a function of radio resource functionality performance. Moreover, realistic operation conditions can present even further variations as a function of time dependent interfering sources, which depend on user behavior, which are specific of the scenario under operation [27].

## 4. Measurements Campaign

### 4.1. Experimental Setup

**Figure 15:**
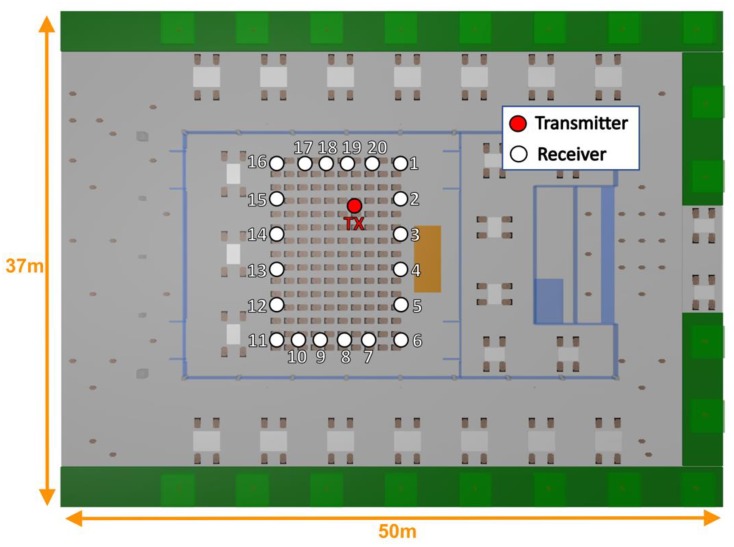
Transmitter position and measurement location points during the measurement campaign for the two analyzed cases: empty and full auditorium.

Validation of previous coverage/capacity estimations have been obtained by means of experimental characterization. A measurement campaign has been performed in the real auditorium placed at university campus at Tecnologico de Monterrey, in Monterrey, Mexico. In order to characterize the losses caused by the presence of different user densities within the auditorium, two different days have been chosen to perform the campaign of measurements: One day with an empty scenario (without people inside and outside of the auditorium) and another day with the full scenario capacity (auditorium full of people due to a conference performance). The transmitter antenna was placed in the same position for both cases, which correspond with the red circle in Figure 15 at 1.2 m height (emulating a transmitter placed in the smart glasses of a seated person). The CC2530 ZigBee development kit, from Texas Instruments, was used as the transmitter, and the N9952A Field Fox portable spectrum analyzer, from Keysight Technologies, as the receiver. The TX and RX antennas were ACA-4HSRPP-2458 from ACKme Networks, both omnidirectional, with a gain of 3.7 dB. Measurements were performed with 10 MHz bandwidth at 2.4 GHz frequency with a measurement time of 60 s at each location point. The received power at each measurement location was considered as the highest peak (Max-Hold function) of power obtained by the spectrum analyzer. Measurements were performed along the complete auditorium, as it is represented in Figure 15. It must be pointed out that the receiver antenna at each measurement location point was placed at the same height of the transmitter, 1.2 m height, which correspond with the head height of a seated person. 

### 4.2. Measurements Results

**Figure 16:**
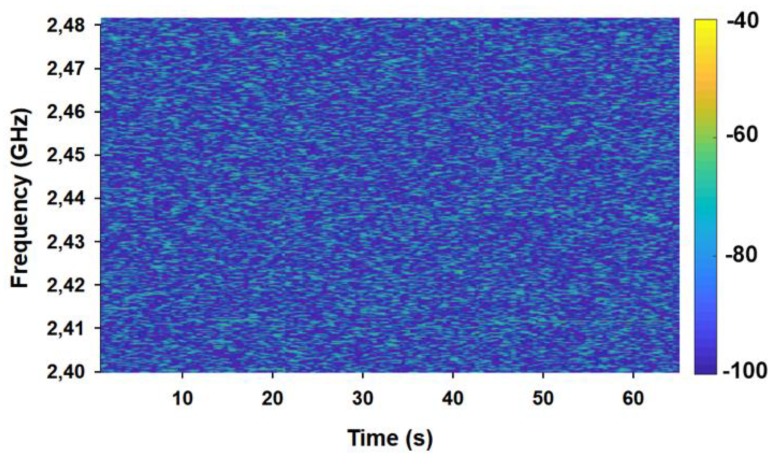
Spectrogram measured before starting the measurement campaign.

In order to assess interference in the frequency band of interest, before starting the measurement campaign for both, empty and full scenario setups, spectrograms were obtained to verify that no other devices were transmitting at the same frequency band of interest that could disturb the measurements. It must be remarked that there were no other wireless systems interference conditioners and the noise level was around −80/−90 dBm, as it can be seen in Figure 16.

**Figure 17:**
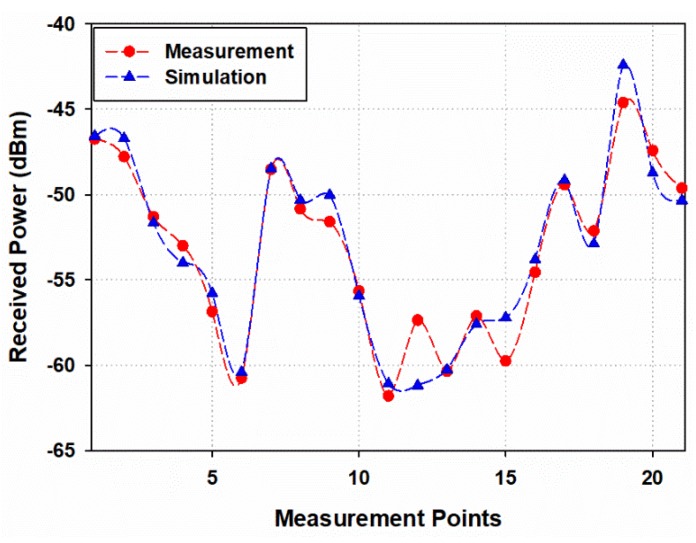
Received power measurements vs 3D ray-launching(3D- RL) simulation comparison.

The most complex or in other words, the worst-case scenario, the auditorium at full user capacity, has been considered for comparison purposes to validate the RL simulation tool. Figure 17 presents the comparison between the 3D-RL simulation tool results and the real measurements in terms of received power for each spatial measurement point represented in Figure 15. It can be seen a good match between simulation and measurements with a mean error of 0.95 dB and standard deviation of 0.92 dB. The differences between simulation and measurement results derive from the fact that the implemented simulation scenario doesn’t consider to a full extent, given by practical limitations, all of the elements within the scenario, with exact shapes, sizes, and the full material set that exists in the real world.

**Figure 18:**
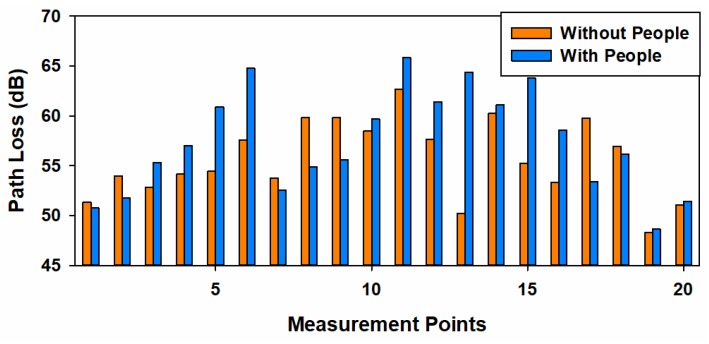
Measured Path Loss for the two measurement cases considered: empty and full scenario.

In addition, in order to get insight into the different losses caused by users’ presence within the considered scenario, Figure 18 presents the path loss comparison for the two different measurement cases, with and without people within the auditorium. The comparison shows that for almost every spatial point the path loss is higher in the full scenario case, due to the absorption caused by the users’ bodies. But this phenomenon depends on the morphology and topology of the scenario, leading to some location points where the path loss is higher in the empty scenario. In average, the path loss in the full scenario (with people) is 3–5 dB bigger than in the empty scenario (without people), which is in accordance with the values reported in [39]. 

## 5. Conclusions and Future Work

In this work, the assessment of different nodes density network configurations has been addressed at 2.4 GHz and 5.8 GHz frequency bands. A complex heterogeneous indoor and outdoor environment has been selected for evaluation, which corresponds with an auditorium placed in a free open city area surrounded by inhomogeneous vegetation. The full analysis of the wireless system in terms of performance and interference characterization has been obtained by means of a deterministic in-house developed 3D-RL algorithm considering different user occupancy. Received signal power as well as SINR have been calculated, showing the effect of degradation when increasing interference in the system. A ZigBee system with O-QPSK modulation has been selected in order to obtain the network performance analysis of the full wireless system setups (infrastructure node network), as well as Bluetooth transceivers in the case of high mobility users. A new processing module has been implemented, enabling the evaluation of modulation constellations and EVM within the complete 3D volume for different indoor and outdoor links setups within the scenario, showing the correct operation regions maps considering different node density cases and distributions. As node density increases, interference values increase considering that radio resource management functionalities are not active, providing a lower bound in terms of coverage/capacity estimations. Moreover, hot-spots can be localized within the scenario, which can strongly impact overall system performance. The sensors placement, individually distributed as well as in mesh setups, is a fundamental parameter in order to assess coverage levels as well as system quality evaluation as a function of SINR. It must be pointed out, that both, coverage estimations analysis and system information provide useful knowledge of the network performance, especially when the number of sensors increases giving rise to high-nodes density scenarios. In addition, a campaign of measurements has been performed in the considered scenario, showing good agreement with simulation results. The proposed methodology makes use of in-house deterministic 3D-RL code, which can consider to a high degree of accuracy elements within the scenario, in terms of shape, size and material characterization. Moreover, the 3D-RL code makes use of hybrid code simulation, employing elements such as neural network interpolators, electromagnetic diffuse scattering or collaborative filtering techniques, which reduce computational complexity and hence, enables the study of large, complex scenarios.

These analysis results and the proposed simulation methodology, can lead in an adequate interference characterization, considering conventional transceivers as well as wearables, which provide suitable information for the overall network performance in complex crowded indoor and outdoor scenarios, with no limitation in scenario definition, following a generalizable approach. Future work will consist in a deeper analysis of the network parameters as well as coverage/capacity analysis for different wireless systems. Besides, the QoS can also be characterized, as well as outage probability. 

## Figures and Tables

**Table 1:** Simulation parameters.

Parameters	Values
Wearable TX Power	4 dBm
Frequency	2.4GHz/5.8 GHz
Bit Rate	250 kbps/1 Mbps/3 Mbps
Antenna Radiation Pattern (RX, TX)/Gain	Omnidirectional/0 dB
3D Ray Launching: Angular Resolution/Reflections	1 degree/6
Scenario size/Unitary volume analysis	(50 × 37 × 8) m/1 m^3^ (1 × 1 × 1) m
System/Modulation/Bandwidth	ZigBee (2.4GHz)/O-QPSK/3 MHz Bluetooth V4.0/8-DPSK/2 MHz
Number of symbols	1000

**Table 2:** Error vector magnitude (EVM) (%) for different nodes-density cases for the ZigBee system.

RMS EVM (%)	High-Node Density	Medium-Node Density	Low-Node Density
Tx 33 (X = 20.35, Y = 22, Z = 1.2) m	32.89	29.6	11.15
Tx 5 (X = 11.7, Y = 3.8, Z = 1.2) m	7.10	3.25	0.0022

**Table 3:** EVM (%) for different nodes-density cases for the Bluetooth V4.0 system.

RMS EVM (%)	High-Node Density	Medium-Node Density	Low-Node Density
Tx 33 (X = 20.35, Y = 22, Z = 1.2) m	47.61	46.39	27.24
Tx 5 (X = 11.7, Y = 3.8, Z = 1.2) m	28.66	11.19	3.06
